# Repurposing ABS to Produce Polyamide 6 (PA6)-Based Blends: Reactive Compatibilization with SAN-g-MA of a High Degree of Functionalization

**DOI:** 10.3390/polym16223103

**Published:** 2024-11-05

**Authors:** Jonathan Vinícius Moreira Torquato, Carlos Bruno Barreto Luna, Edson Antonio dos Santos Filho, Emanuel Pereira do Nascimento, Tomás Jeferson Alves de Mélo, Renate Maria Ramos Wellen, Edcleide Maria Araújo, Dayanne Diniz de Souza Morais

**Affiliations:** 1 Laboratory of Composites and Structural Integrity and Laboratory of Biocorrosion and Corrosion, Department of Mechanical Engineering, Center for Technology and Geosciences, Federal University of Pernambuco, Recife 50740-550, PE, Brazil; 2 Academic Unit of Materials Engineering, Federal University of Campina Grande, Av. Aprígio Veloso, 882-Bodocongó, Campina Grande 58429-900, PB, Brazil; 3 Department of Materials Engineering, Federal University of Paraíba, Cidade Universitária, João Pessoa 58051-900, PB, Brazil

**Keywords:** ABSr, reuse, polymer blends, polyamide 6, reactive compatibilization

## Abstract

In this study, recycled acrylonitrile-butadiene-styrene terpolymer (ABSr) was reused to produce polyamide 6 (PA6)-based blends. This was achieved through reactive compatibilization using styrene-acrylonitrile-maleic anhydride (SAN-g-MA) copolymer with a high degree of functionalization (6–10% MA). The PA6/ABSr and PA6/ABSr/SAN-g-MA blends were prepared through melt processing and injection molding and then analyzed for their rheological, mechanical, thermomechanical, thermal, and structural properties, as well as morphology. The torque rheometry revealed a maximum reactivity of the PA6/ABSr (70/30 wt%) blend with low SAN-g-MA (5 phr—parts per hundred resin) content, while above this threshold, torque began to decline, indicating compatibilizer saturation in the interface. These findings were further substantiated by the increase in complex viscosity and the lower melt flow index (MFI) of the PA6/ABSr/SAN-g-MA (5 phr) blend. The 5 phr SAN-g-MA reactive compatibilization of the PA6/ABSr blends significantly enhanced its impact strength, elongation at break, tensile strength, and heat deflection temperature (HDT) by 217%, 631%, 12.6%, and 9.5%, respectively, compared to PA6/ABSr. These findings are promising for the plastic recycling field, paving the way for the production of new tailor-made materials at a reduced price.

## 1. Introduction

Various products in the plastics processing sector are made from synthetic polymer materials, meeting the most diverse functional requirements both economically and effectively [1,2]. On the other hand, the growing demand for polymer-based products aggravates environmental and social problems, especially in large urban centers [3,4]. This phenomenon is caused by the low density of traditional polymers and their resistance to biological degradation, which makes them occupy vast spaces in the environment for a long time [5,6]. Ecological and economic solutions for the final disposal of polymer waste have been encouraged in the industrial sector, environmental organizations, and other social segments [7,8]. Open-air landfill disposal is not a definitive solution for waste polymer-based products, as the sun, rain, wind, and micro-organisms can degrade plastic and form plastic fragments [9,10]. These factors combined can lead to the formation of unwanted by-products, such as microplastics, generating contamination in the water, soil, and air [11]. Therefore, plastic waste reuse, both from industrial processes and from products discarded by society, has been significantly established through mechanical recycling [12,13,14].

Recycling, as an essential action for sustainable development, plays a significant role in incorporating post-consumer material into the production chain [15]. The profitability of the recycling market has increased, encouraging, and benefiting companies in the polymer industry sector. This, in turn, has positive socio-economic repercussions on the life quality of the population, including income generation, natural resource conservation, and reduced environmental problems [16,17]. These aspects stimulated the addition of polymer residues into new formulations with virgin resins, which is presented in the literature as an economically advantageous and sustainable way to produce polymer blends [18,19,20]. Polymer blends are highly important for the plastics processing sector, mainly because they allow the design of new materials with improved characteristics compared to neat polymers, using processing and molding techniques widely acknowledged in the industry [20,21]. Manufacturers of polymer blends are producing new materials with a fraction of recycled material in the formulations, which contributes to sustainable development, enables the reinsertion of recycled material into the production chain, and adds value [22,23]. From this perspective, the post-consumer acrylonitrile-butadiene-styrene (ABS) terpolymer deserves attention in polymer blends since this engineering material is widely used in the automotive industry [24,25].

ABS is an amorphous terpolymer with processing stability, facile injection molding, superior impact strength at low temperatures, and a high heat deflection temperature (HDT) [26]. It is widely used in the automotive industry to make instrument panels, lamp holders, column covers, grilles, and mirror housings [27]. As such, the disposal of ABS-derived products has grown markedly, primarily because of its increased use in additive manufacturing (3D printing) [28,29]. Therefore, the technological potential of ABS makes it possible to engineer polymer blends with recycled ABS, producing new materials with tailored mechanical and thermomechanical properties and increasing the range of applications. The outstanding features of ABS have led to its use as an impact modifier for polyamide 6 (PA6), as reported in the literature [30,31,32,33]. However, PA6/ABS blends are regarded as immiscible and incompatible [34]. Thus, PA6/ABS blends have poor mechanical properties as the high interfacial tension and low interfacial adhesion between phases lead to premature fracture under mechanical impact stress. For this reason, reactive compatibilizing agents containing maleic anhydride are often added to stabilize the blend morphology, improve interfacial adhesion, and enhance the mechanical properties of PA6/ABS blends [35,36,37,38,39].

Oliveira et al. [40] melt processed PA6/ABS blends using styrene-maleic anhydride (SMA) as a compatibilizing agent. The PA6/ABS blend showed poorer impact strength than PA6, suggesting increased brittleness and low molecular interaction between its components. The addition of 5% SMA to the PA6/ABS blend promoted a reaction between the PA6 amine end groups and maleic anhydride, increasing the viscosity of the compatibilized blend. The PA6/ABS/SMA (57.5/37.5/5 wt%) system increased impact strength by 59% and HDT by more than 15 °C compared to PA6. The elastic modulus remained practically unchanged from that of PA6, suggesting that SMA successfully made PA6/ABS compatible. Ren et al. [41] compatibilized PA6/ABS blends with styrene-acrylonitrile-graft-maleic anhydride (SAN-g-MA). The SAN-g-MA compatibilizer improved interaction and interfacial adhesion within the PA6/ABS blends. In addition, the size of the ABS phase was refined in the PA6 matrix, helping to improve the mechanical properties: impact strength, tensile strength, flexural strength, and elongation at break.

The SAN-g-MA copolymer has been incorporated into PA6/ABS blends because the similarity between the SAN phase and ABS favors their interaction. Additionally, maleic anhydride may interact with the amine end groups of PA6, improving the mechanical properties of the final product [42]. However, the scarcity of research on the making of PA6 blends with recycled ABS underlines the need for experimental reports to enrich the literature database. Reusing recycled polymer is of utmost importance for the environmental, economic, and social sectors, hence research focusing on understanding the properties and potential applications of recycled polymer is needed. The challenge is to find the optimum composition to improve mechanical properties with recycled materials as part of the composition. In general, recycled material contains contaminants that impair the final performance of the polymer blend developed.

The aim of the present research was to develop polyamide 6 (PA6) blends with recycled ABSr from automotive mirror housings. In addition, styrene-acrylonitrile-graft-maleic anhydride (SAN-g-MA) with a high degree of grafting (6–10% MA) was used as a compatibilizing agent for the PA6/ABSr blend to improve the mechanical properties.

## 2. Materials and Methods

### 2.1. Materials

The polyamide 6 (PA6) polymer matrix (specification B300), in pellets, with a density of 1.13 g/cm^3^, was supplied by Thathi Polymers (São Paulo, Brazil). Flake-like acrylonitrile-butadiene-styrene (ABSr, where “r” means recycled) came from automobile mirror carcass waste provided by the plastic recycling industry in Campina Grande, Brazil. The styrene-acrylonitrile-graft-maleic anhydride copolymer (SAN-g-MA) pellets, with 6–10% maleic anhydride (MA), and the code Coace^®^ KS-03, with a melt flow index of 5–15 g/10 min (ASTM D1238–200 °C/5 kg) and density 1.09 g/cm^3^ (ASTM D792), were supplied by Coace Chemical Company Limited (Beijing, China).

### 2.2. Preparation and Molding of Polymer Blends

PA6, ABSr, and SAN-g-MA materials were dried for 24 h in a vacuum oven set at 80 °C before melt processing to remove moisture. The PA6/ABSr and PA6/ABSr/SAN-g-MA blends were melt processed in a Haake Rheomix 3000 torque rheometer (Thermo Scientific Polylab QC, Waltham, MA, USA), with a volume of 310 cm^3^ and using 70% of its capacity. Roller-type rotors operating at 60 rpm and a temperature of 240 °C were used during processing. The residence time was 10 min. The materials processed in the internal mixer were crushed in a knife mill before injection molding. The designed compositions were PA6/ABSr (70/30 wt%), PA6/ABSr + SAN-g-MA (70/30 wt% + 5 phr), and PA6/ABSr + SAN-g-MA (70/30 wt% + 10 phr). SAN-g-MA was used as a compatibilizing agent in the PA6/ABSr blend and added in parts per hundred of resin (phr). For comparison, the neat materials were subjected to the same processing conditions as the polymer blends.

**Figure 1:**
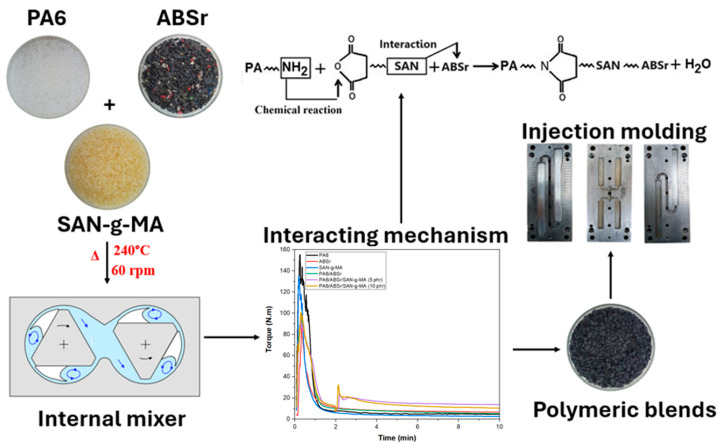
Process route used to obtain the polymer blends.

Before injection molding, the crushed materials were dried in vacuum ovens (80 °C/24 h). Subsequently, the test specimens were injected using an Arburg injection molding machine, model Allrounder 207C Golden Edition (Arburg Inc., Loßburg, Germany). The injection molding operation parameters were injection pressure of 1200 bar, holding pressure of 800 bar, mold temperature of 50 °C, mold cooling time of 25 s, and temperature profile of 230, 240, 240, 240, and 250 °C. The molded samples were tested for tensile strength, impact strength, Shore D hardness, and heat deflection temperature (HDT), following recommendations from ASTM D638, ASTM D256, ASTM D2240, and ASTM D 648, respectively. Figure 1 shows the processing flowchart used to obtain the polymer blends.

### 2.3. Characterization of Polymer Blends

The rheology analysis in the oscillatory regime was carried out using an Anton Paar rheometer (Anton Paar AG, Graz, Austria), model MCR 301, equipped with a 25 mm diameter parallel plate geometry. The experimental parameters were air atmosphere, a temperature of 240 °C, a gap between plates of 1 mm, and a frequency ranging from 0.1 to 600 rad/s. The deformation adopted was 1% within the linear viscoelasticity region. Samples from the impact test were used during experiments.

SAN-g-MA compatibilizer reactivity with PA6 and ABSr was analyzed in a torque rheometer (Thermo Scientific Polylab QC, Waltham, MA, USA), operating at 240 °C and 60 rpm during 10 min of melt processing.

The melt flow index (MFI) was determined using a Hebert Lambert extrusion plastometer, model CLP Schneider 3210 (Hebert Lambert, São Paulo, Brazil). The analysis was conducted according to ASTM D1238 standard at 235 °C and 2.16 kg of load. It was performed on an average of ten samples.

Fourier transform infrared spectroscopic (FTIR) analysis was performed on a Bruker Spectrometer (Bruker Corporation, Billerica, MA, USA), model Alpha II, using the ATR (attenuated total reflectance) method. Spectra were collected by scanning the surface of injected samples (thickness of 3.2 mm) in the 4000 to 400 cm^−1^ range, with a resolution of 4 cm^−1^ and 32 scans per sample.

The Molau test consisted of dissolving 2 g of neat PA6, SAN-g-MA, and the PA6/SAN-g-MA (90/10%) blend in 40 mL of formic acid (85%) under magnetic stirring.

The Izod impact strength was determined at room temperature using notched specimens in Ceast Resil 5.5 type equipment (Instron, Norwood, MA, USA), with a 2.75 J pendulum (ASTM D256 standard). The analysis was evaluated using an average of ten samples.

The Shore D hardness test was carried out with Metrotokyo equipment (MetroTokyo, São Paulo, Brazil), following the ASTM D2240 standard. A load of 50 N was applied to the sample at ten random points, and the indenter was pressed for 10 s.

Tensile strength was measured using universal Oswaldo Filizola BME equipment (Oswaldo Filizola Ltda, São Paulo, Brazil), following the ASTM D638 recommendations. The test was conducted at room temperature with a 5 mm/min deformation speed and a load cell of 20 kN. The results were evaluated with an average of ten samples.

Differential scanning calorimetry (DSC) was conducted on Shimadzu equipment (Shimadzu Corporation, Kyoto, Japan), model DSC-60Plus. During analyses, nitrogen was employed as the carrier gas (50 mL/min), and 2.5–2.8 mg samples were subjected to a heating-cooling-heating program from 50 to 250 °C, employing a 10 °C/min heating rate. The determination of the degree of crystallinity (Xc) was calculated using Equation (1) [42,43]:

(1) $$X_{c}=\frac{{∆H}_{f}}{w*{∆H}_{f}}\times 100\%$$

where ∆H_f_ = crystalline melting enthalpy obtained by DSC; w = mass fraction of PA6; ∆H_f 100%_ = melting enthalpy of PA6 with 100% crystallinity, 190.8 J/g [43,44].

The heat deflection temperature (HDT) analysis was carried out on CEAST equipment (Instron, Norwood, MA, USA), model HDT 6 VICAT, following the recommendations of the ASTM D648 standard. Test conditions were a heating rate of 120 °C/h, a load of 1.82 Mpa, and a sample deflection of 0.25 mm. Silicone oil was used as the immersion medium, wherein three samples were analyzed simultaneously for average purposes.

The morphology of the neat polymers and polymer blends was evaluated on the fracture surface of the impact test using a VEGAN scanning electron microscope (SEM), model MIRA LMU (TESCAN, Brno, Czech Republic), at a voltage of 5 keV and high vacuum. Prior to analysis, the surfaces of the samples were goldsputtered.

## 3. Results and Discussion

### 3.1. Dynamic-Oscillatory Rheology

**Figure 2:**
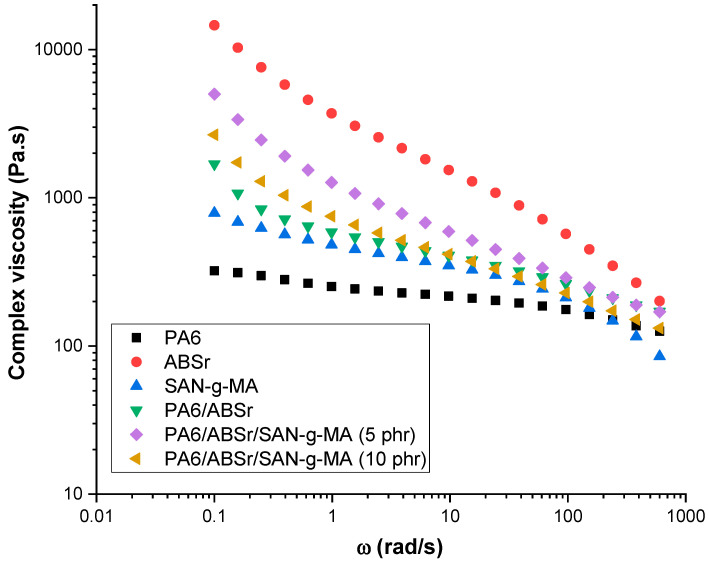
Complex viscosity curves for PA6, ABSr, SAN-g-MA, and polymer blends.

Dynamic oscillatory rheology provides data on the viscous and elastic properties of neat polymers and polymer blends at low frequencies. The data of this regime are sensitive to minor differences in structure and are therefore relevant to studying the effect of SAN-g-MA on PA6/ABSr. Figure 2 shows the complex viscosity plots as a function of angular frequency (ω) for neat polymers and the polymer blends with and without the SAN-g-MA compatibilizer. In the 0.1–100 rad/s range, neat PA6 showed the lowest viscosity amidst the materials analyzed. In addition, a Newtonian fluid-like behavior was observed, with the viscosity being independent of frequency. Contrastingly, ABSr exhibited a pseudoplastic fluid behavior, as the complex viscosity decreased with the angular frequency. SAN-g-MA exhibited a hybrid behavior, behaving like a Newtonian fluid at frequencies below 10 rad/s while showing a pseudoplastic character above this value. The PA6/ABSr and PA6/ABSr/SAN-g-MA blends exhibited intermediate behavior between the neat polymers regarding complex viscosity, predominantly tending towards a pseudoplastic fluid behavior. SAN-g-MA compatibilization of the PA6/ABSr blend slightly impacted the rheological profile, as seen in Figure 2. For frequencies up to 10 rad/s, the PA6/ABSr/SAN-g-MA systems showed increased complex viscosity compared to PA6/ABSr. This behavior may be due to the SAN/ABSr interaction and a reaction between the maleic anhydride groups of SAN-g-MA and the amine terminal groups of PA6 [45]. This assumption is supported by the complex viscosity results, as the PA6/ABSr formulation remained fixed at 70/30 wt%. SAN-g-MA had a lower complex viscosity compared to the non-compatibilized blend, which could have caused a reduction in complex viscosity, but it did not occur. The complex viscosity increment of the PA6/ABSr/SAN-g-MA blends reflects the improved compatibility between phases fostered by SAN-g-MA, leading to higher molecular intertwining and, consequently, higher flow resistance. These events ultimately contributed to an increase in complex viscosity.

The complex viscosity reveals a different behavior in the PA6/ABSr/SAN-g-MA blends promoted by the effect of the SAN-g-MA. Adding 5 phr of SAN-g-MA to the PA6/ABSr blend increased the complex viscosity, uncovering interactions between chemical groups. However, the complex viscosity decreased for a higher SAN-g-MA content (10 phr). Apparently, the 10 phr of SAN-g-MA was in excess in the PA6/ABSr blend, especially considering a compatibilizer with a high degree of functionalization (6–10% g-MA). This can be attributed to the limited number of amine functional groups in the PA6 chains available for reaction. Therefore, possibly not all maleic anhydride reacted with the amine groups of PA6, forming an excess that acted as a lubricant and reduced the complex viscosity of the PA6/ABSr/SAN-g-MA blend (10 phr). The PA6/ABSr/SAN-g-MA (5 phr) formulation was enough to promote a more robust molecular entanglement, improving the mechanical properties.

Figure 2 shows that as the shear rate increased (above 100 rad/s), the neat polymers and polymer blends converged to a similar complex viscosity. As shear rates increase, the polymer chains unwind and orient themselves in the flow direction, reducing viscosity.

**Figure 3:**
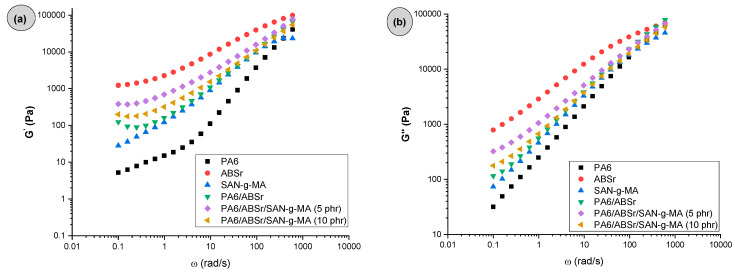
Elastic and dissipative response curves of the neat polymers and polymer blends: (**a**) storage modulus; (**b**) loss modulus.

Figure 3 presents the storage modulus (G′) and loss modulus (G″) as a function of angular frequency (ω) for neat polymers and polymer blends. The G′ component is related to elastic behavior, while G″ is due to viscous dissipation [46]. Below 10 rad/s, G′ and G″ values of PA6/ABSr/SAN-g-MA blends increased compared to the PA6 and the PA6/ABSr blend, demonstrating that SAN-g-MA increased the viscoelastic characteristics of the PA6/ABSr/SAN-g-MA blends. Figure 3a,b shows that the PA6/ABSr/SAN-g-MA blends, in the 0.1–10 rad/s range, had G′ and G″ values intermediate to the neat polymers; although, they were superior to that of the PA6/ABSr blend. In addition, the PA6/ABSr/SAN-g-MA blends presented a slope in the low frequencies (<10 rad/s) tending towards zero, indicating a pseudo-solid behavior. Their curves surpass that of PA6/ABSr, indicating higher compatibility in the presence of SAN-g-MA. The oscillatory rheological analysis is sensitive to evaluate the modification of polymer blends by incorporating a compatibilizer. For instance, below 1 rad/s, the addition of SAN-g-MA increased the G′ curve for the PA6/ABSr/SAN-g-MA blends in relation to the PA6/ABSr system. Such behavior suggests that interactions between SAN-g-MA and PA6/ABSr increased the elastic response. This effect is likely due to a higher level of molecular entanglement in the PA6/ABSr/SAN-g-MA blend, which results in an increased G′. The increase in viscoelastic behavior was more prominent for PA6/ABSr/SAN-g-MA (5 phr), suggesting the formation of a more entangled molecular structure [47]. This finding shows a rise in the elastic response of the PA6/ABSr/SAN-g-MA (5 phr) blend, corroborating the elongation at break shown later. The viscous response (G′′) follows the same trend as G′. Again, the 5 phr SAN-g-MA content proved to be a critical concentration to maximize the viscoelastic response of the PA6/ABSr blend, corroborating the hypothesis that 10 phr of SAN-g-MA is excessive and harmed the performance of the polymer blend.

**Figure 4:**
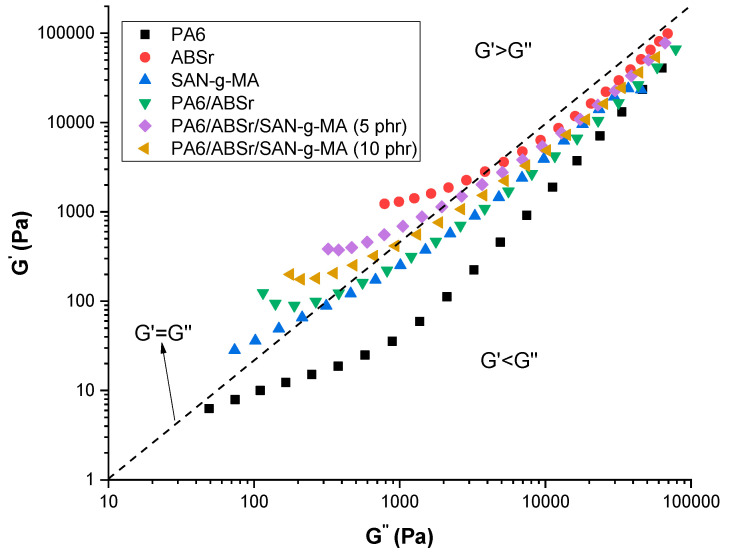
Storage modulus (G′) versus loss modulus (G″) for the neat polymers and polymer blends as a function of SAN-g-MA content.

Figure 4 presents the G′ versus G″ graph for neat polymers and polymer blends, with and without the SAN-g-MA compatibilizer, to evaluate the viscous and elastic behavior. The G′ = G″ line divides the graph into two parts: above the line (G′ > G″), where the material presents a more elastic than viscous behavior; and below the line (G′ < G″), where the material presents a more viscous behavior [48,49]. The PA6 matrix had a viscous fluid behavior, considering that its curve remains below the G′ = G″ (G′ < G″ region). ABSr and SAN-g-MA transitioned from elastic to viscous behavior, especially in the 10–1000 Pa (G″) range. The PA6/ABSr and PA6/ABSr/SAN-g-MA blends also exhibited an elastic-viscous transition, more pronounced for PA6/ABSr/SAN-g-MA (5 phr). In the 10–1000 Pa (G″) range, the polymer blends are in G′ > G″, a predominant elastic response, while above 1000 Pa (G″), it changed to a more viscous behavior. Incorporating SAN-g-MA into the PA6/ABSr blend increased the G′ curve to a higher value than PA6/ABSr, especially at 5 phr. Consequently, the elastic character of the PA6/ABSr/SAN-g-MA (5 phr) blend was stronger, which is directly reflected in the improved impact strength and elongation at break, as discussed later.

**Figure 5:**
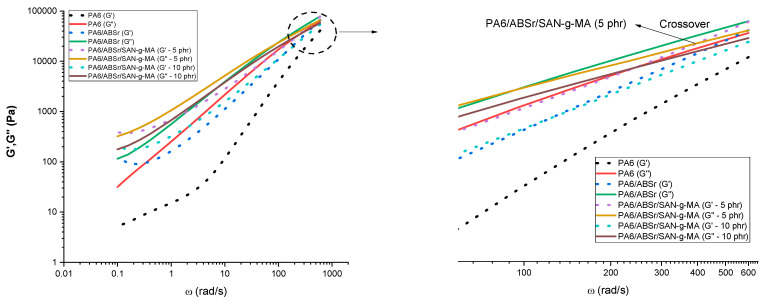
G′ and G″ curves as a function of frequency for PA6 and polymer blends with and without the SAN-g-MA compatibilizer.

Figure 5 shows the behavior of the G′, G″ graphs as a function of angular frequency for PA6, the PA6/ABSr system, and the PA6/ABSr/SAN-g-MA blends. The displacement of the crossover when G′ (ɷ) coincides with G″ (ɷ) makes it possible to predict the increase or decrease in molar mass (MM) [50,51].

It was noted that in the whole frequency range of 0.1–1000 rad/s, neat PA6, PA6/ABSr, and the PA6/ABSr/SAN-g-MA (10 phr) blend did not present a crossover between G′ and G″. PA6/ABSr/SAN-g-MA (5 phr) was the only system that presented a crossover point, which suggests a higher level of molecular entanglement, directly producing higher viscosity. According to Figure 5, the crossover occurred at G′ = G″ = 5.3 × 10^4^ Pa and ɷ = 396 rad/s. Therefore, the PA6/ABSr/SAN-g-MA (5 phr) blend had a higher molar mass, probably due to the better balance of maleic anhydride. This result agrees with the complex viscosity and the mechanical properties discussed later.

### 3.2. Torque Rheometry

**Figure 6:**
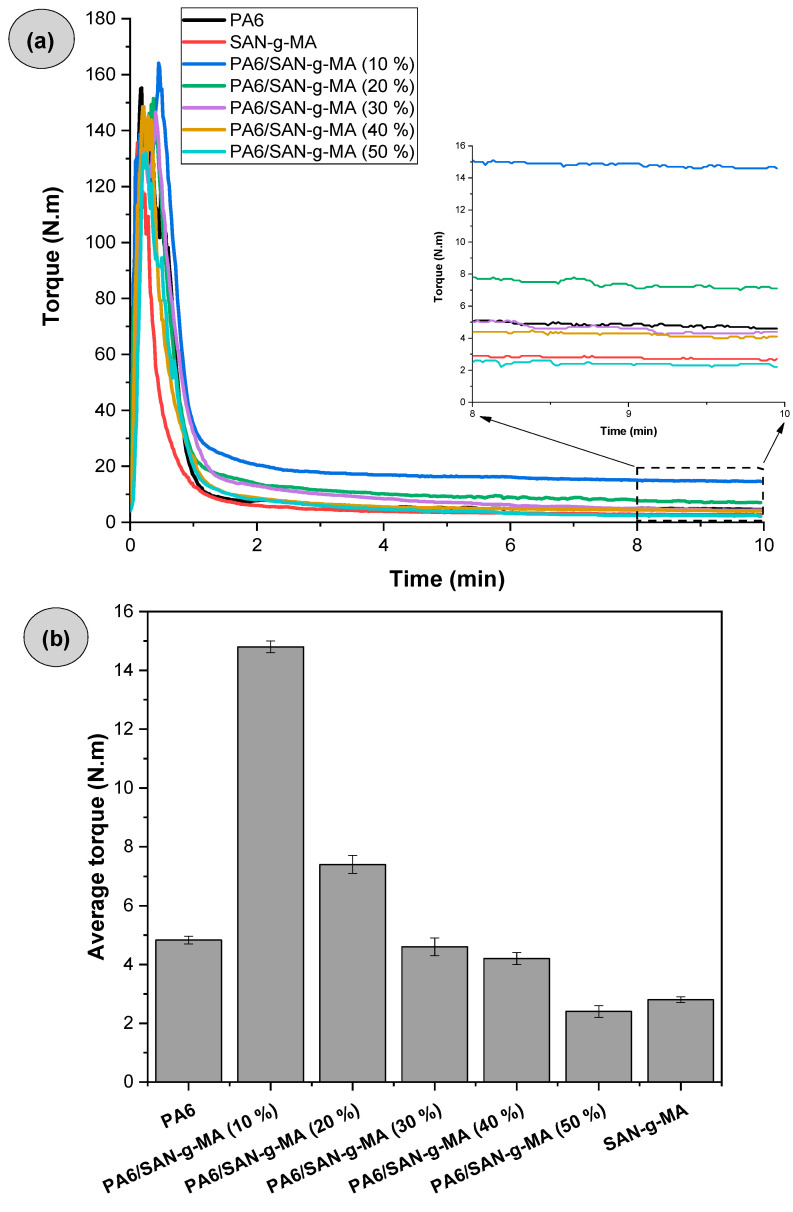
Torque rheometry of PA6, SAN-g-MA, and PA6/SAN-g-MA blends. (**a**) Curves of reactivity as a function of time. (**b**) Average stabilized torque in the 8–10 min interval.

Torque rheometry analysis is essential to investigate the chemical interactions between PA6 and maleic anhydride functionalized copolymers. Torque rheometry curves may signal that the functionalized copolymer is in excess in the PA6, which may adversely affect the mechanical properties. Figure 6a presents the torque versus time curves of PA6, the SAN-g-MA copolymer, and the PA6/SAN-g-MA blends. The average torque results in the 8–10 min interval are reported in Figure 6b. Torque rheometry was initially performed on PA6/SAN-g-MA blends to confirm whether there was a reaction between maleic anhydride and the amine terminal groups of PA6. The investigation was carried out with an increasing fraction of SAN-g-MA (10–50 wt%) in PA6 to understand the impact of increasing maleic anhydride content on reactivity.

**Figure 7:**
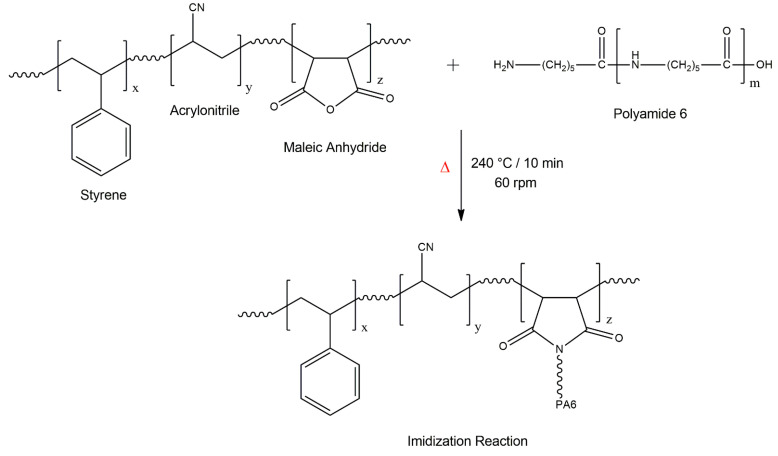
Reaction mechanism between PA6 and SAN-g-MA, forming the imide group. The symbol Δ represents heating.

During melt processing in the internal mixer, the terminal torque curve of polymers and polymer blends is directly proportional to viscosity (η) [52,53]. Figure 6a shows a maximum torque peak associated with the dissipation of mechanical energy during the compaction of the solid material. Subsequently, the torque tended to reduce and stabilize, given the plasticization of the material. In this case, the material melts and sustains the flow in the mixing chamber, suggesting that the torque is proportional to viscosity. The stabilized torques of PA6 and SAN-g-MA were 4.8 N.m and 2.7 N.m, respectively. The PA6/SAN-g-MA (10%) blend showed a noteworthy increase in the stabilized average torque to about 14.8 N.m, surpassing PA6 and SAN-g-MA. It suggests a viscosity increment for the PA6/SAN-g-MA (10%) blend promoted by an increased molecular entanglement. This behavior was possibly caused by the reaction between the maleic anhydride in SAN-g-MA and the amine terminal groups of PA6, as shown in Figure 7. According to the literature [45,54], the maleic anhydride groups react with the terminal amine groups of polyamide 6, forming an imide group and resulting in an in situ copolymer at the interface. However, as the SAN-g-MA content increased, a constant decrease in the stabilized average torque was observed, being more noticeable in the PA6/SAN-g-MA (50%) blend. Therefore, it is possible to infer that there is a critical concentration of a high-functionalized reactive compatibilizer to improve the interaction with PA6 because there is a limited amount of terminal amine groups in the PA6 chain. For the 10% SAN-g-MA content, a balanced reaction level with PA6 produced a higher viscosity. In contrast, the functional groups were excessive in the 20–50% SAN-g-MA range. In other words, the amine groups of PA6 reacted with the maleic anhydride of SAN-g-MA, while the excess can no longer react and works as a lubricant, reducing torque and viscosity [55].

**Figure 8:**
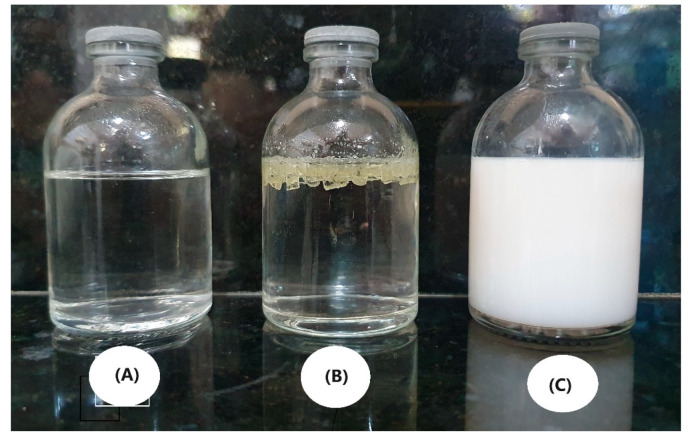
Molau test in formic acid for: (**A**) PA6; (**B**) SANg-g-MA; (**C**) PA6/SAN-g-MA blend (90/10%).

The Molau test was applied to investigate whether there was an interaction between PA6 and the SAN-g-MA compatibilizer, as shown in Figure 8. The PA6/SAN-g-MA (10%) blend was selected because it presented the highest torque value, suggesting a better reaction efficiency between the amine groups and maleic anhydride. The literature [56,57] has suggested that the Molau test effectively evaluates the reactive compatibilization of PA6-based blends using functionalized copolymers. In general, when a reaction between functional groups occurs, the solution formed becomes whitish due to the emulsion of the reacted material. Figure 8A shows a clear solution, indicating that neat PA6 dissolved in formic acid. On the other hand, SAN-g-MA did not dissolve in formic acid, and the granules precipitated on the surface. The PA6/SAN-g-MA blend formed a white solution, suggesting a reaction between the amine terminal groups and maleic anhydride, corroborating the torque rheometry. This behavior occurred because polyamide 6 dissolved in formic acid and dragged the SAN-g-MA phase, which reacted, generating a white solution. In this way, it is possible to notice strong evidence of the formation of a graft copolymer, as shown in the mechanism in Figure 7.

**Figure 9:**
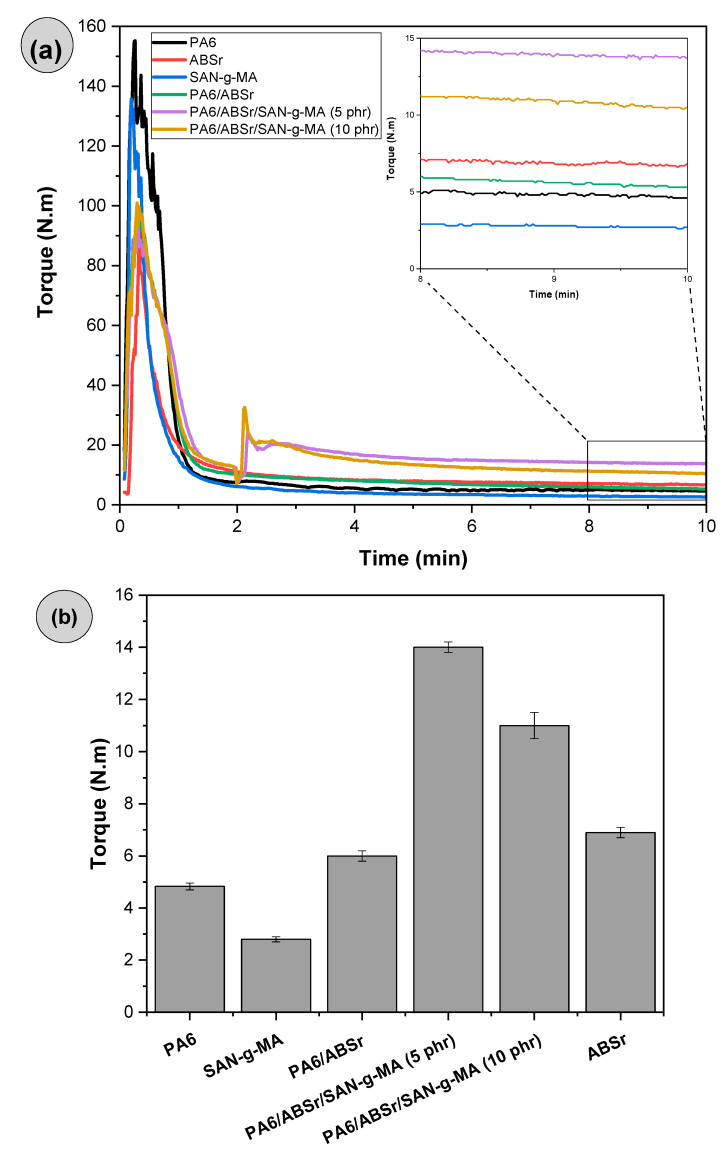
Torque rheometry of neat polymers and polymer blends: (**a**) torque versus times curves; (**b**) average stabilized torque in the 8–10 min region.

Figure 9a presents the torque versus time curves for PA6, SAN-g-MA, and polymer blends with and without compatibilizer. The average torque values obtained between 8–10 min are shown in Figure 9b.

Neat PA6 and SAN-g-MA presented the lowest torque values, while ABSr had a higher torque value (6.9 N.m). The PA6/ABSr blend showed a torque intermediate between PA6 and ABSr, with a value of 6 N.m. In this case, the additivity effect prevailed, i.e., torque increased due to the 30% ABSr added, which is more viscous. The torque of the PA6/ABSr/SAN-g-MA blends had a notable increase, surpassing the neat polymers. This finding corroborates the hypothesis that SAN-g-MA reacted with the terminal amine groups of PA6 and interacted with ABSr. The 5 phr SAN-g-MA content was more efficient at compatibilizing the PA6/ABSr, achieving the highest torque. The robust torque increase for the PA6/ABSr/SAN-g-MA (5 phr) blend suggests the amount of maleic anhydride was ideal for reacting with PA6, forming chains with higher molar mass and, consequently, higher viscosity. Although the torque also increased for the PA6/ABSr/SAN-g-MA (10 phr) blend, the 10 phr amount is probably in excess, generating a lubricating effect and causing a deleterious outcome on viscosity when compared to 5 phr SAN-g-MA. Except for ABSr, the torque rheometry results agree with the complex viscosity. According to Figure 2, ABSr presented the highest complex viscosity among the prepared materials, which was not reflected in a higher torque. This phenomenon indicates that ABSr was more sensitive to processing under a higher shear rate, resulting in an inverse viscosity behavior. The parallel plate rheometry measures low shear rates, while the internal mixer high-intensity rotors (roller) generate high shear rates.

### 3.3. Melt Flow Index (MFI)

**Figure 10:**
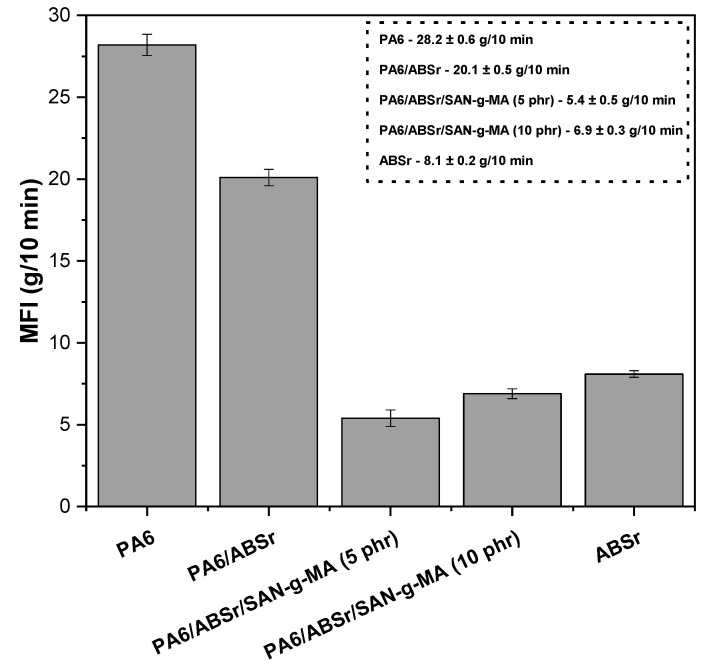
Melt flow index of PA6, ABSr, and polymer blends with and without SAN-g-MA.

The melt flow index (MFI), a widely used method in the polymer industry for its simplicity and quick determination, was employed to measure the flow resistance of PA6, ABSr, and polymer blends, as depicted in Figure 10.

PA6 presented an MFI of 28.2 g/10 min, the highest among the compositions, indicating a lower flow resistance and, therefore, a lower viscosity. The ASTM D1238 standard recommends 200 °C/5 kg or 220 °C/10 kg as the MFI test conditions for ABS. However, ABSr was tested using the same parameters as PA6 for comparative purposes. ABSr presented an MFI of 8.2 g/min, suggesting a higher flow resistance than neat PA6. The PA6/ABSr blend exhibited an MFI of 20.1 g/10 min. Therefore, adding 30% ABSr increased the blend’s viscosity while keeping a high fluidity level. The addition of SAN-g-MA to the PA6/ABSr blend, regardless of its content, caused a notable decrease in MFI. This indicates that SAN-g-MA increased the molecular entanglement, allowing a higher degree of interaction between the molecular segments of SAN and ABSr and between maleic anhydride and the amine groups of PA6. The PA6/ABSr/SAN-g-MA (5 phr) blend showed the lowest MFI (5.4 g/10 min), probably due to the increase in molar mass, corroborating torque rheometry. Again, the 10 phr SAN-g-MA content reduced viscosity compared to the 5 phr compatibilizer, supporting the notion that maleic anhydride is in excess and acts as a lubricant.

### 3.4. Fourier Transform Infrared Spectroscopy (FTIR)

**Figure 11:**
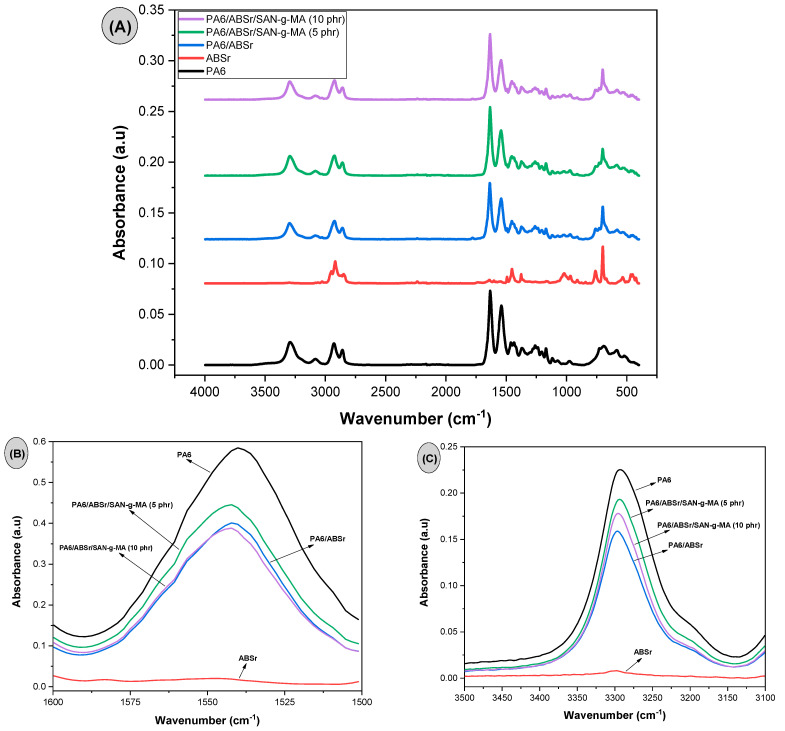
FTIR spectra of PA6, ABSr, and the polymer blends, with scans of: (**A**) 4000 to 400 cm^−1^; (**B**) 1600 to 1500 cm^−1^; (**C**) 3500 to 3100 cm^−1^.

Figure 11A–C presents the infrared spectra of PA6, ABSr, and polymer blend with and without SAN-g-MA. The Supplementary Materials (Supplementary File S1) presents the infrared spectra of ABSr, commercial ABS, PP and PE, evidencing that ABSr is contaminated with PP and PE.

The infrared spectrum of PA6 showed bands at 3290 cm^−1^, 3072 cm^−1^, 2926 cm^−1^, and 2855 cm^−1^ corresponding to the stretching vibration of the N-H group, stretching vibration of the C-H bond, and the asymmetric and symmetric stretching of methylene (CH_2_) [58], respectively. The intense bands at 1634 cm^−1^ and 1538 cm^−1^ are ascribed to Amide I (stretching vibration of the C=O group) and Amide II (stretching of the CN groups and bending of NH) [59], respectively. The γ crystalline phase is present in PA6 due to bands at 1435 cm^−1^ (bending vibration of the CH_2_ group close to the NH group) and 976 cm^−1^ (in-plane CO-NH). Furthermore, the band close to 1200 cm^−1^ is attributed to the α crystalline phase of PA6 [60]. Accordingly, PA6 presented both α and γ crystalline phases, corroborating the DSC results presented later. PA6/ABSr and PA6/ABSr/SAN-g-MA blends exhibited the main vibrational bands of PA6. However, the intensity of the 3290 cm^−1^ and 1538 cm^−1^ bands reduced, as shown in Figure 11B,C, which was probably caused by the PA6 reduction in the formulations (30%). The intensity of the 3290 cm^−1^ and 1538 cm^−1^ features was higher for the PA6/ABSr/SAN-g-MA (5 phr) blend compared to the PA6/ABSr and other PA6/ABSr/SAN-g-MA formulations. A higher interaction between SAN-g-MA and the PA6/ABSr blend was observed for 5 phr SAN-g-MA, which was probably due to an adequate amount of maleic anhydride available to react with the amine groups of PA6. This phenomenon caused the formation of stronger bands at 3290 cm^−1^ and 1538 cm^−1^ for PA6/ABSr/SAN-g-MA (5 phr), which concur with torque rheometry.

### 3.5. Scanning Electron Microscopy (SEM)

**Figure 12:**
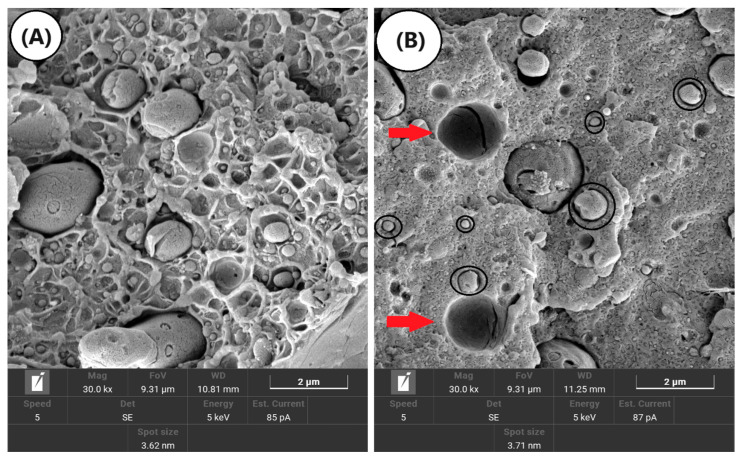

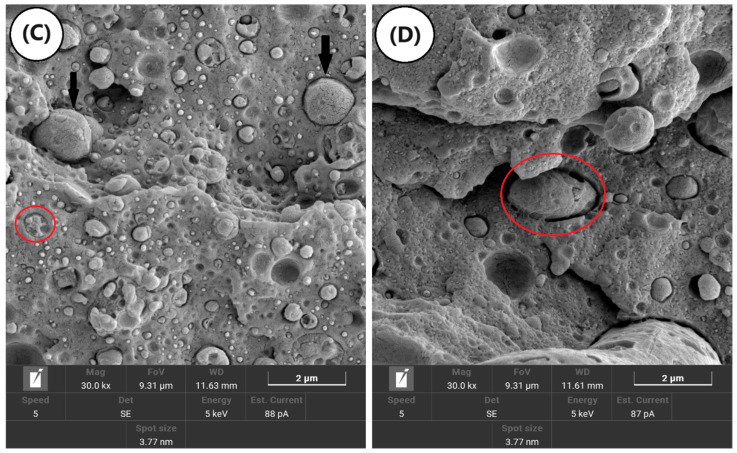
Evolution of the morphology of ABSr and polymer blends with and without SAN-g-MA: (**A**) ABSr; (**B**) PA6/ABSr; (**C**) PA6/ABSr/SAN-g-MA (5 phr); (**D**) PA6/ABSr/SAN-g-MA (10 phr).

Figure 12A–D shows the Izod impact fractured surface of ABSr, the PA6/ABSr system, and the PA6/ABSr/SAN-g-MA blends. Figure 12A shows that ABSr has a heterogeneous morphology, with dispersed particles of varying sizes, indicating the presence of contaminants. This morphology agrees with the FTIR since ABSr showed vibrational bands typical of polypropylene and polyethylene (see Supplementary File S1). The difference in the molecular structure of these contaminants (PP and PE) and ABSr produced a typical morphology of an immiscible and incompatible blend, with low adhesion at the interface caused by differences in interfacial tension. As reported later, ABSr displayed low impact and tensile mechanical properties because of these contaminations.

The PA6/ABSr blend presented a morphology with distinct phases (Figure 12B), showing spherical ABSr particles dispersed in the PA6 matrix and a heterogeneous particle size distribution. Furthermore, the PA6 matrix presented voids (red arrows) with a smooth internal surface, indicating low adhesion and high interfacial tension between PA6 and ABSr. The evidence suggests a low resistance interface that does not favor an effective stress transfer between PA6 and ABSr phases, which explains the low mechanical properties. The black circles indicate that several ABSr particles had no interfacial interaction with PA6, which is typical of an immiscible blend. Under mechanical stress conditions, the lack of interfacial interaction causes microvoids at the interface, leading to premature fracture. A notable feature in the PA6/ABSr morphology is the onset of inclusion cavitation inside the ABSr particle, resulting from the PP and PE contaminants.

The SAN-g-MA was added to PA6/ABSr to evaluate the effect of compatibilization on the mechanical properties, especially impact strength and elongation at break. The compatibilizer increases adhesion between phases, reducing interfacial tension and preventing the coalescence between particles of the minority phase [61,62]. Figure 12C showed that the PA6/ABSr/SAN-g-MA (5 phr) blend had a more stable morphology with a better distribution of the dispersed phase. Clearly, some large dispersed particles are present, though the number of refined particles predominated. According to torque rheometry and MFI, the PA6/ABSr/SAN-g-MA (5 phr) blend presented a higher viscosity, which probably improved the shear stress transfer to the dispersed phase, increasing particle rupture. The PA6/ABSr/SAN-g-MA (5 phr) blend reveals a hybrid morphology with particles well-adhered to the PA6 matrix and, at the same time, particles with low adhesion. The compatibilization of the PA6/ABSr/SAN-g-MA (5 phr) blend was satisfactory since broken spherical particles were observed in the PA6 matrix (see red circle). This observation suggests that the interface did not rupture during the impact test; thereby, the interfacial adhesion between PA6 and ABSr improved when SAN-g-MA was added. Figure 12C clearly shows the existence of very small particles, which could also be the SAN-g-MA forming a third phase dispersed in the PA6 matrix. Refining the size of the dispersed phase is relevant to the mechanical properties of polymer blends, as it increases the interfacial area, consequently improving stress transfer. The scientific literature [63,64] demonstrates that satisfactory toughening of polyamide 6 can be achieved with dispersed phase sized between 0.1–2 μm. The PA6/ABSr/SAN-g-MA (5 phr) blend presented a significant number of particles between 0.1–2 μm (according to the SEM scale), although some particles larger than 2 μm were seen (see black arrows). This morphology explains the highest recovery in impact strength and elongation at break among the polymer blends. PA6/ABSr/SAN-g-MA (10 phr) (Figure 12D) formed a coarser morphology compared to PA6/ABSr/SAN-g-MA (5 phr). Partial compatibility was evidenced by some particles showing low interfacial adhesion, while others did not cavitate and sustained deformation. As highlighted by the red circle in Figure 12D, a ligament is present at the interface between PA6 and ABSr, indicating that part of the SAN-g-MA migrated to the interface, generating interfacial resistance. However, the 10 phr SAN-g-MA content did not promote an adequate distribution of the dispersed phase in the PA6 matrix, given the small amount in the analyzed area and the presence of large particles. Apparently, the higher maleic anhydride content promoted coalescence in the PA6/ABSr/SAN-g-MA (10 phr) blend, reflected by the larger particle size compared to the blend compatibilized with 5 phr SAN- g-MA.

### 3.6. Impact Strength

**Figure 13:**
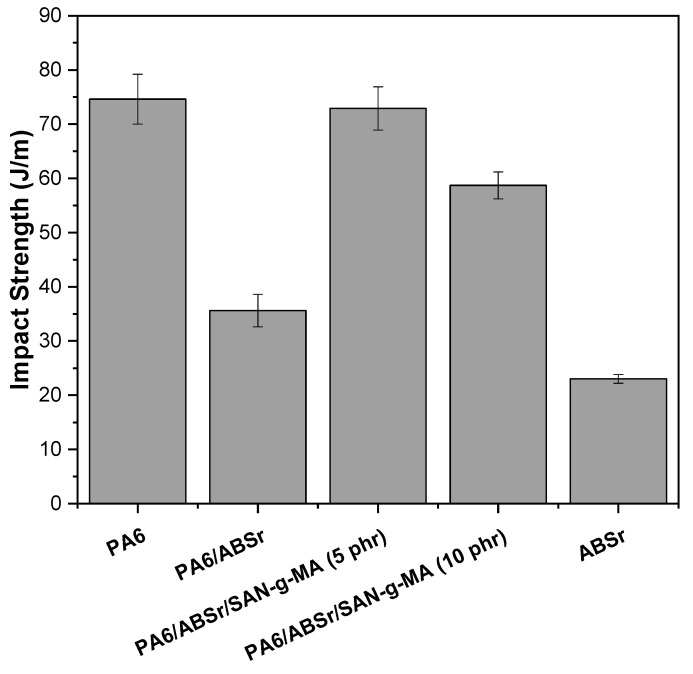
Mechanical behavior under impact for PA6, ABSr, and polymer blends.

Figure 13 presents the Izod impact strength of neat PA6, ABSr, and PA6/ABSr blends with and without SAN-g-MA.

Neat PA6 showed an impact strength of 74.6 J/m, consistent with the literature [65]. In contrast, ABSr presented an impact strength of 23 J/m, a value much lower than the average for high-impact commercial ABS of 390–450 J/m [66,67]. This discrepancy, as revealed by FTIR and SEM analyses, could be attributed to the contamination of ABSr with polypropylene and polyethylene, which likely contributed to its brittleness upon impact. The addition of ABSr as an impact modifier to PA6 was ineffective, even having a deleterious effect, as 30% ABSr significantly reduced the impact strength of PA6. This finding further confirmed the low interfacial interaction between PA6 and ABSr suggested by SEM analysis. This behavior may be ascribed to the distinct molecular structure of PA6 and ABSr, which caused interfacial tension and yielded an immiscible and incompatible PA6/ABSr blend. The lack of interfacial interaction causes microvoids at the interface of the PA6/ABSr blend, which leads to crack formation and facilitates their propagation, resulting in low impact strength [68,69]. Therefore, adding a compatibilizing agent to this system is crucial. A similar result had already been reported in another study [70], where the compatibilization of the PA6/ABSr blend with SAN-g-MA promoted positive changes in the behavior under impact.

The PA6/ABSr/SAN-g-MA (5 phr) blend presented the highest impact strength, with a value of 72.9 J/m. In this case, the impact properties are comparable to neat PA6, considering the experimental error. The impact strength of the formulation with 5 phr SAN-g-MA is approximately 217% higher than that of the uncompatibilized blend. The data supports the assumption that SAN-g-MA acted as a compatibilizing agent for PA6/ABSr, which likely resulted from the miscibility of SAN-g-MA with ABSr, and the reaction of maleic anhydride with the amine terminal groups of PA6 [42]. Consequently, SAN-g-MA could stabilize the morphology of the PA6/ABSr blend, reducing interfacial tension and improving adhesion, improving stress transfer. In general, 5 phr of SAN-g-MA was sufficient to toughen the PA6/ABSr blend, being the optimal concentration to promote synergism in the impact property. Contrastingly, the 10 phr SAN-g-MA concentration promoted a deleterious effect on impact strength compared to PA6/ABSr/SAN-g-MA (5 phr), representing an excess of compatibilizer. As the complex viscosity demonstrated, PA6 and ABSr interaction optimization occurred at 5 phr SAN-g-MA content, yielding higher impact strength compatibility. Increasing SAN-g-MA from 5 to 10 phr probably induced saturation of maleic anhydride in the PA6/ABSr blend, generating stress concentration and reducing the impact strength. This behavior can be attributed to the limited number of amine functional groups in the PA6 molecular structure. Therefore, adding a large amount of SAN-g-MA to the PA6/ABSr blend to guarantee an efficient interaction is unnecessary because there are not enough amine terminal groups to react. In fact, torque curves of the PA6/SAN-g-MA blends demonstrated that a lower SAN-g-MA content maximized the torque, indicating that a low concentration was enough to compatibilize the blend. A similar result was found in the literature [54] for PA6/AES blends compatibilized with MMA-MA, wherein an excess maleic anhydride in MMA-MA reduced the impact strength.

### 3.7. Tensile Strength

**Figure 14:**
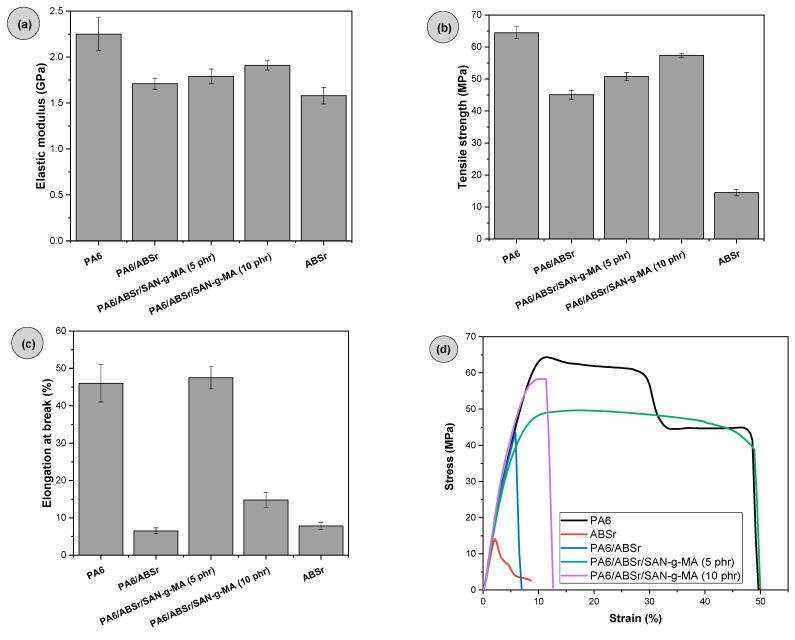
Mechanical behavior under tensile stress for PA6, ABSr, and polymer blends: (**a**) elastic modulus; (**b**) tensile strength; (**c**) elongation at break; (**d**) stress–strain curves.

Figure 14a–d shows the elastic modulus, tensile strength, elongation at break, and the stress–strain curves. Neat PA6 presented higher resistance to elastic deformation, reflected in a robust elastic modulus of 2.4 GPa. The literature [71] indicates that the elastic modulus for PA6 is within the 1.1–3.2 GPa range. ABSr presented an elastic modulus of 1.58 GPa, lower than that of PA6, due to the presence of the flexible butadiene groups. PA6/ABSr and PA6/ABSr/SAN-g-MA blends showed elastic moduli intermediate to the respective neat polymers, reflecting the additivity effect. The addition of 30% ABSr in the PA6 matrix made the elastic modulus reach 1.71 GPa, a value 28.7% lower than that of neat PA6. On the contrary, reducing PA6 concentration by adding a flexible material into the formulation decreased the elastic modulus. The SAN-g-MA compatibilizer combined with the PA6/ABSr blend promoted a moderate recovery in the elastic modulus. For example, the PA6/ABSr/SAN-g-MA (5 phr) blend underwent a 5% increase in elastic modulus compared to PA6/ABSr (although with no statistically significant differences). On the other hand, the PA6/ABSr/SAN-g-MA (10 phr) system induced an 11.7% recovery and showed a more prominent stiffness among the polymer blends produced in this work. These PA6/ABSr/SAN-g-MA blends showed an increased stiffness, yet not enough to reach that of neat PA6. Increasing the SAN-g-MA content from 5 to 10 phr, i.e., doubling the amount of the SAN fraction, contributed to increasing the PA6/ABSr/SAN-g-MA blend stiffness.

PA6 showed the highest tensile strength (64.5 MPa), needing the highest tensile load to undergo deformation. In contrast, ABSr had a low tensile strength, suggesting that PP and PE contaminants acted as stress concentrators, inducing premature fracture. Compared to neat PA6, the PA6/ABSr blend reduced tensile strength by 30%. The tensile strength property is determined in the plastic region and consequently depends on an acceptable interfacial adhesion to withstand maximum stress. Therefore, if the interaction between PA6 and ABSr is insufficient, microvoids will form at the interface, facilitating crack formation and propagation, ultimately reducing the tensile strength. Thus, the SAN-g-MA compatibilizer had a positive improving effect on the tensile strength of PA6/ABSr. The PA6/ABSr/SAB-g-MA (5 phr) and PA6/ABSr/SAB-g-MA (10 phr) blends provided increments in tensile strength of 12.6% and 27.2%, respectively, compared to PA6/ABSr. These data further support the idea that SAN-g-MA acts as a compatibilizing agent within the PA6/ABSr system, improving stress transfer at the interface. The intermediate tensile strength values of the PA6/ABSr/SAB-g-MA (5 phr) and PA6/ABSr/SAB-g-MA (10 phr) blends, compared to neat components, indicate a good interfacial adhesion between phases, as highlighted in the SEM analysis.

Elongation at break is a property that depends on appropriate interaction between components in a polymer blend. Otherwise, the fracture will be brittle and without ductility. PA6 and ABSr elongated by 46% and 7.8%, respectively. ABSr could not improve the ductility of PA6, considering that the elongation at break of the PA6/ABSr blend reduced to 6.5%. The significant decrease in the elongation at break compared to neat PA6 reveals the incompatibility of the PA6/ABSr blend. The SAN-g-MA compatibilizer was incorporated into the PA6/ABSr blend to improve its ductility. Adding 5 phr SAN-g-MA to the PA6/ABSr/SAN-g-MA blend remarkably improved the elongation at break by 631% compared to the uncompatibilized PA6/ABSr blend. Furthermore, the ductility recovered to a level analogous to neat PA6, considering the experimental error (see Figure 14c). The higher molecular entanglement provided an increase in elongation at break. The rheology tests showed that SAN-g-MA could react with the terminal amine groups of PA6 and provided a satisfactory interaction between SAN and the ABSr phase, consequently improving the elongation at break. However, the 5 phr SAN-g-MA was the threshold value to efficiently compatibilize the PA6/ASBRr blend. The 10 phr of compatibilizer led to a drop in elongation at break. The elongation of the PA6/ABSr/SAN-g-MA (10 phr) blend increased by 127.6% compared to PA6/ABSr. Nevertheless, this value was lower than those of PA6 and the PA6/ABSr/SAN-g-MA (5 phr) blend, which indicates that 10 phr SAN-g-MA is in excess, harming the flexibility level.

Figure 14d shows that the stress–strain curve of PA6 has a high tensile stress and ductility, while ABSr shows the behavior of brittle material with a low deformation level. The PA6/ABSr blend kept the ABSr fragility, showing low deformation and confirming the poor PA6 and ABSr interaction and incompatibility. There was an outstanding ductility recovery for the PA6/ABSr/SAN-g-MA (5 phr) blend, which showed a ductility level comparable to neat PA6. This behavior indicates a high level of molecular intertwining between PA6 and ABSr fostered by the 5 phr SAN-g-MA, which promoted a synergic effect within the blend. In addition, the tensile strength slightly increased, indicating strengthening at the interface. Incorporating 10 phr of SAN-g-MA resulted in a more prominent increase in tensile strength, although with a reduction in deformation compared to the PA6/ABSr/SAN-g-MA (5 phr) blend. Increasing SAN-g-MA content from 5 to 10 phr caused an increase in maleic anhydride and the SAN phase, which produced a decline in the degree of mobility.

### 3.8. Shore D Hardness

**Figure 15:**
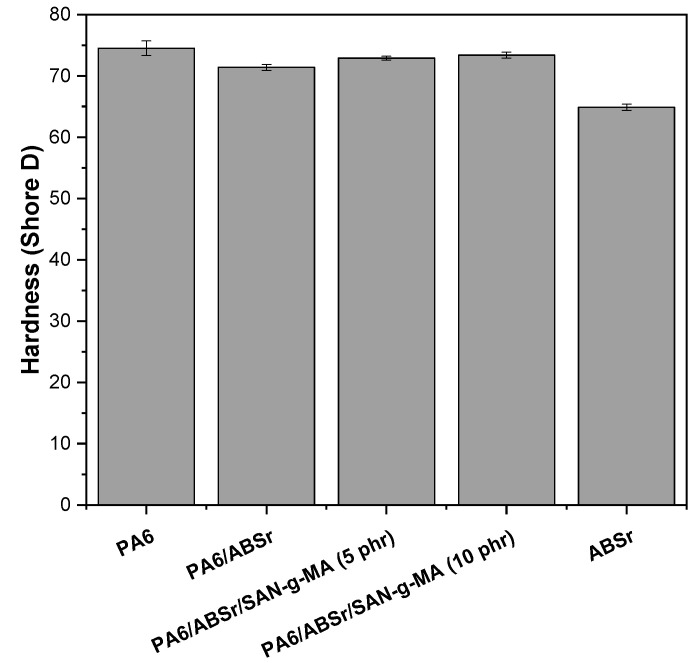
Shore D hardness of PA6, ABSr, and polymer blends as a function of SAN-g-MA content.

The Shore D hardness property is widely used in the plastic industry to measure the polymer resistance to surface penetration. Figure 15 shows the Shore D hardness behavior of PA6, ABSr, and polymer blends with different SAN-g-MA contents.

Neat PA6 exhibited the highest hardness level with 74.5 Shore D. This result shows a higher resistance to penetration, probably caused by the higher stiffness of PA6, as shown by its elastic modulus. In contrast, ABSr had the lowest hardness performance, with a 64.9 Shore D value. This behavior is owed to the presence of butadiene in the molecular structure of ABSr, forming a surface that is more flexible and less resistant to penetration. On the other hand, the PA6/ABSr and PA6/ABSr/SAN-g-MA blends presented an intermediate behavior between the neat polymers, i.e., they suffered an additivity effect. Adding 30% ABSr to the PA6 matrix slightly decreased the Shore D hardness by 6%. The SAN-g-MA compatibilizer added to the PA6/ABSr blend recovered the Shore D hardness to values close to PA6. The compatibilizer is composed of hard styrene-acrylonitrile, contributing to increasing rigidity and, consequently, the Shore D hardness of the PA6/ABSr/SAN-g-MA blends. In addition, the hardness of compositions containing 5 phr and 10 phr SAN-g-MA showed no statistical differences, suggesting that the PA6/ABSr/SAN-g-MA (5 phr) formulation was sufficient to optimize the Shore D hardness. Even though the 10 phr SAN-g-MA contains a higher amount of styrene-acrylonitrile, it did not tend to present higher Shore D hardness values than the composition with 5 phr SAN-g-MA.

### 3.9. Heat Deflection Temperature (HDT)

**Figure 16:**
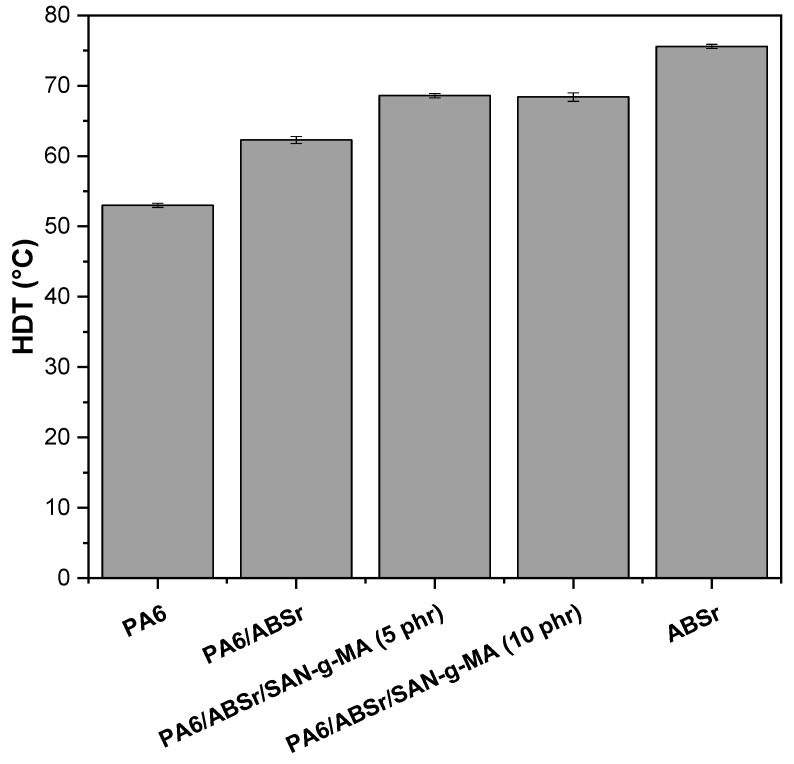
Heat deflection temperature of PA6, ABSr, and polymer blends.

HDT analysis is very relevant for plastics applications, as it indicates structural stability, i.e., the resistance to deformation of the polymer at higher temperatures [72,73]. The HDT results of PA6, ABSr, and polymer blends with and without the SAN-g-MA compatibilizer are reported in Figure 16.

PA6 and ABSr presented HDT in the order of 53.4 °C and 75.6 °C, respectively, indicating that ABSr has high thermomechanical stability. The significant HDT of ABSr is attributed to the SAN phase, which provides rigidity and thermal and chemical resistance. The PA6/ABSr and PA6/ABSr/SAN-g-MA blends show higher HDT than neat PA6 but with lower performance than neat ABSr. This behavior indicates that the additivity effect influenced the HDT of polymer blends, i.e., the gain was due to the dispersed fraction of ABSr in PA6. The 30% ABSr incorporated into the PA6 matrix increased the HDT value to 62.4 °C, corresponding to a 16.8% increment compared to neat PA6. Although ABSr is a recycled material, it showed a high performance at increasing the thermomechanical resistance of PA6. Similar behavior was reported in other investigations [40,74] concerning PA6/ABSr blends, however, using virgin ABS. Adding 5 and 10 phr of SAN-g-MA copolymer into the PA6/ABSr (70/30%) blend helped to understand the HDT behavior in this fixed proportion. The PA6/ABSr/SAN-g-MA (5 phr) and PA6/ABSr/SAN-g-MA (10 phr) systems further improved the HDT value to 68.3 °C and 68.6 °C, respectively, compared to neat PA6 and the uncompatibilized blend. The SAN-g-MA copolymer in the PA6/ABSr blend promoted a higher increase in thermomechanical resistance by around 6 °C compared to PA6/ABSr. PA6/ABSr blend composition was fixed at 70/30%. Thus, the HDT increase is likely the result of higher SAN concentration in the PA6/ABSr/SAN-g-MA blend formulation that created higher thermomechanical resistance. The PA6/ABSr/SAN-g-MA (5 phr) and PA6/ABSr/SAN-g-MA (10 phr) blends presented comparable HDT results, considering the experimental error. In this case, compatibilization of the PA6/ABSr blend, regarding the HDT property, was achieved with 5 phr SAN-g-MA, and the 10 phr copolymer represents an excess. This finding denotes a crucial economic factor since a lower SAN-g-MA concentration can be used to achieve compatibilization.

PA6 has a limitation concerning its low heat deflection temperature, restricting its applications where mechanical load and temperature are used simultaneously. Therefore, adding 30 wt% of recycled material effectively improved the HDT of PA6, which is a promising result for the reintroduction of ABSr in the plastics transformation chain.

### 3.10. Differential Scanning Calorimetry (DSC)

**Figure 17:**
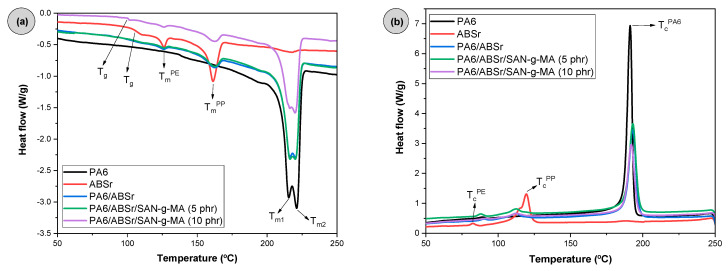
DSC curves of PA6, ABSr, and polymer blends as a function of SAN-g-MA content. (**a**) second heating cycle; (**b**) crystallization.

Figure 17 and Table 1 present the melting and crystallization parameters, especially the crystalline melting temperature (T_m_), crystallization temperature (T_c_), glass transition (T_g_), and the degree of crystallinity (X_c_) of PA6, ABSr, and polymer blends. The DSC curves are those obtained during the second heating cycle. The reuse of recycled material is complex due to the heterogeneity of its composition, as demonstrated for ABSr. It was observed that ABSr presented its glass transition from the SAN phase at around 107 °C. According to Figure 17a, ABSr presented crystalline melting temperatures of 162 °C and 126 °C, suggesting the presence of polypropylene and polyethylene [75,76], respectively. This confirms the FTIR result of ABSr (see Supplementary File S1). The PA6 matrix presented two crystalline melting temperature events, designated as T_m1_ and T_m2_. T_m1_ corresponds to the appearance of the least thermally stable phase around 215.6 °C. This peak, as reported in the literature [77], is related to the formation of the γ crystalline phase in PA6. The temperature peak around 221.2 °C concerns the α phase of neat PA6 [78]. The PA6/ABSr and PA6/ABSr/SAN-g-MA blends maintained the crystalline melting events of PA6 and ABSr, without major significant changes. However, the glass transition temperature (T_g_) of the PA6/ABSr blend decreased to 104.7 °C compared to ABSr. The introduction of SAN-g-MA more prominently reduced the T_g_ of the PA6/ABSr/SAN-g-MA blends to the range of 101–102 °C. This behavior suggests an increase in the mobility of the PA6/ABSr/SAN-g-MA blends compared to the PA6/ABSr system, corroborating the impact strength and elongation at break.

Figure 17b shows two crystallization temperatures (T_c_) for ABSr, related to the polyethylene and polypropylene fractions. PA6 had a very intense T_c_ peak at 191.2 °C, analogous to that found by Zhang et al. [79]. The PA6/ABSr and PA6/ABSr/SAN-g-MA blends slightly increased the T_c_ value compared to neat PA6. Furthermore, the intensity of the T_c_ peaks of the polymer blends decreased since the amount of PA6 in the formulation was reduced. Regarding the degree of crystallinity, the highest value of 28.5% was obtained for PA6. The addition of ABSr negatively affected the degree of crystallinity of the PA6/ABSr and PA6/ABSr/SAN-g-MA blends, indicating no nucleating agent effect. The PA6/ABSr/SAN-g-MA (5 phr) blend presented a value close to that of PA6, probably contributing to the high mechanical tensile results and Shore D hardness. The reduction was more drastic for the PA6/ABSr/SAN-g-MA (10 phr), which coincides with the deterioration of mechanical properties compared to PA6/ABSr/SAN-g-MA (5 phr). This finding suggests that an excess of SAN-g-MA negatively impacted the degree of crystallinity, reducing the crystallinity to 23.5%.

## 4. Conclusions

In this work, ABSr from car mirror carcass waste was reused to make polymer blends based on PA6, aiming at manufacturing new tailored materials. The results showed that ABSr reuse is complex because the composition is heterogeneous. Therefore, the direct mixture of PA6/ABSr presented low mechanical properties, given the intrinsic immiscibility and incompatibility. One of the ways to solve this problem is to incorporate low concentrations of SAN-g-MA with a high degree of functionality into the PA6/ABSr blend. This finding is essential to cost reduction since the functionalized compatibilizer has a high added value. Therefore, the ABSr and low SAN-g-MA content combination was an acceptable option for obtaining materials with tailored mechanical properties close to PA6. The PA6/ABSr/SAN-g-MA (5 phr) formulation promoted a synergism of properties, with gains in impact strength, elongation at break, tensile strength, and heat deflection temperature (HDT) of 217%, 631%, 12.6%, and 9.5%, respectively, compared to PA6/ABSr. Furthermore, ABSr can improve the thermomechanical stability of PA6, increasing PA6 resistance to deformation under mechanical and thermal stress. The results presented are relevant to the plastics processing industry since the recycled ABSr can potentially be reused to manufacture components for the electrical sector. Polymer blend formulations with recycled material contribute to sustainability by minimizing environmental damage and maintaining performance and competitive prices.

## Figures and Tables

**Table 1:** Thermal parameters of PA6, ABSr, and polymer blends.

Samples	T_g_ (°C)	T_m_^PA6^ (°C)		T_c_^PA6^ (°C)	X_c_^PA6^ (%)
		T_m1_ (°C)	T_m2_ (°C)		
PA6	-	215.6	221.2	191.3	28.5
ABSr	107.0	-	-	-	-
PA6/ABSr	104.7	216.3	220.3	193.1	26.8
PA6/ABSr/SAN-g-MA (5 phr)	101.2	216.3	220.2	193.2	28.0
PA6/ABSr/SAN-g-MA (10 phr)	101.8	216.1	220.1	192.1	23.5

## Data Availability

Data are contained within the article.

## Supplementary Materials

The following supporting information can be downloaded at: https://www.mdpi.com/article/10.3390/polym16223103/s1, Supplementary File S1: Fourier-transform infrared spectroscopy (FTIR); Figure S1: FTIR spectra of ABSr, commercial ABS, PP and PE; Figure S2: FTIR spectra of ABSr, commercial ABS, PP and PE.
