# Supplementary material for: Repurposing ABS to Produce Polyamide 6 (PA6)-Based Blends: Reactive Compatibilization with SAN-g-MA of a High Degree of Functionalization
Source: Polymers (Basel). 2024 Nov 5;16(22):3103. doi: 10.3390/polym16223103 (PMC11597962; doi:10.3390/polym16223103)
Supplement: Supplementary file 1 [file polymers-16-03103-s001.zip › polymers-3205850-supplementary.pdf]

Repurposing ABS to Produce Polyamide 6 (PA6)-Based Blends: Reactive Compatibilization with SAN-g-MA of a High Degree of Functionalization

Jonathan Vinícius Moreira Torquato[1], Carlos Bruno Barreto Luna[2], Edson Antonio dos Santos Filho[2], Emanuel Pereira do Nascimento[2], Tomás Jeferson Alves de Mélo[2], Renate Maria Ramos Wellen[3], Edcleide Maria Araújo[2] and Dayanne Diniz de Souza Morais[1]*

[1] Laboratory of Composites and Structural Integrity and Laboratory of Biocorrosion and Corrosion, Department of Mechanical Engineering, Center for Technology and Geosciences, Federal University of Pernambuco, Recife – PE, 50740-550, Brazil

[2]Academic Unit of Materials Engineering, Federal University of Campina Grande, Av. Aprígio Veloso, 882 - Bodocongó, 58429-900, Campina Grande - Paraíba, Brazil.

[3]Department of Materials Engineering, Federal University of Paraíba, Cidade Universitária, 58051-900, João Pessoa, PB, Brazil.

* To whom correspondence should be addressed: dayanne.diniz@ufpe.br

Supplementary File S1 - Fourier-transform infrared spectroscopy (FTIR)

Fourier-transform infrared spectroscopy (FTIR) analysis was performed on a Bruker Spectrometer, model Alpha II, using the ATR (attenuated total reflectance) method. The test was conducted on the surface of injected samples with a thickness of 3.2 mm, using a scan of 4000 to 400 cm^{-1}, with a resolution of 4 cm^{-1} and 32 scans.

The FTIR spectra of ABSr, commercial ABS, PP, and PE are presented in supplementary material 1. The ABSr spectrum showed a typical band at 2236 cm^{-1}, referring to the acrylonitrile group. In addition, bands of polypropylene (PP) and polyethylene (PE) are observed, suggesting that it is a heterogeneous recycled material.

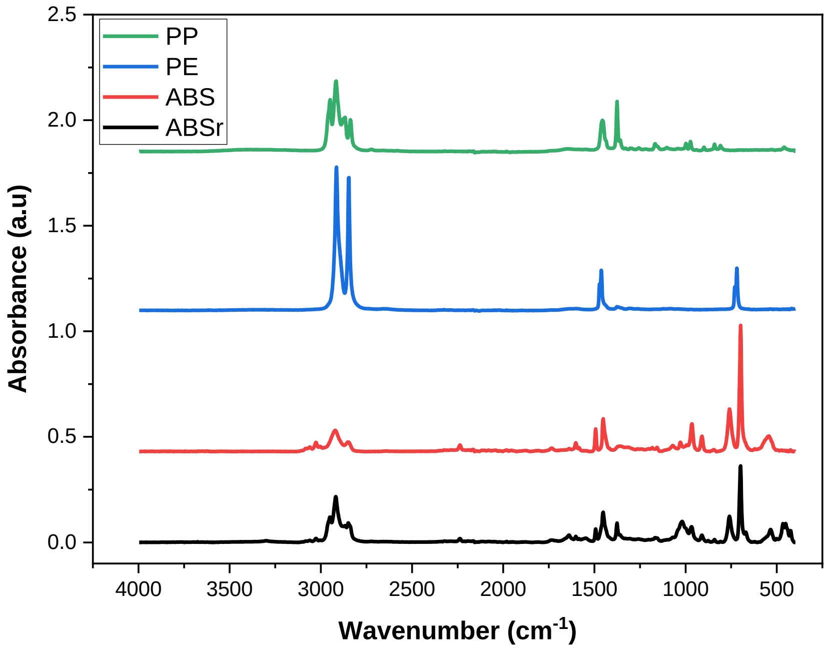

Figure S1. FTIR spectra of ABSr, commercial ABS, PP and PE.

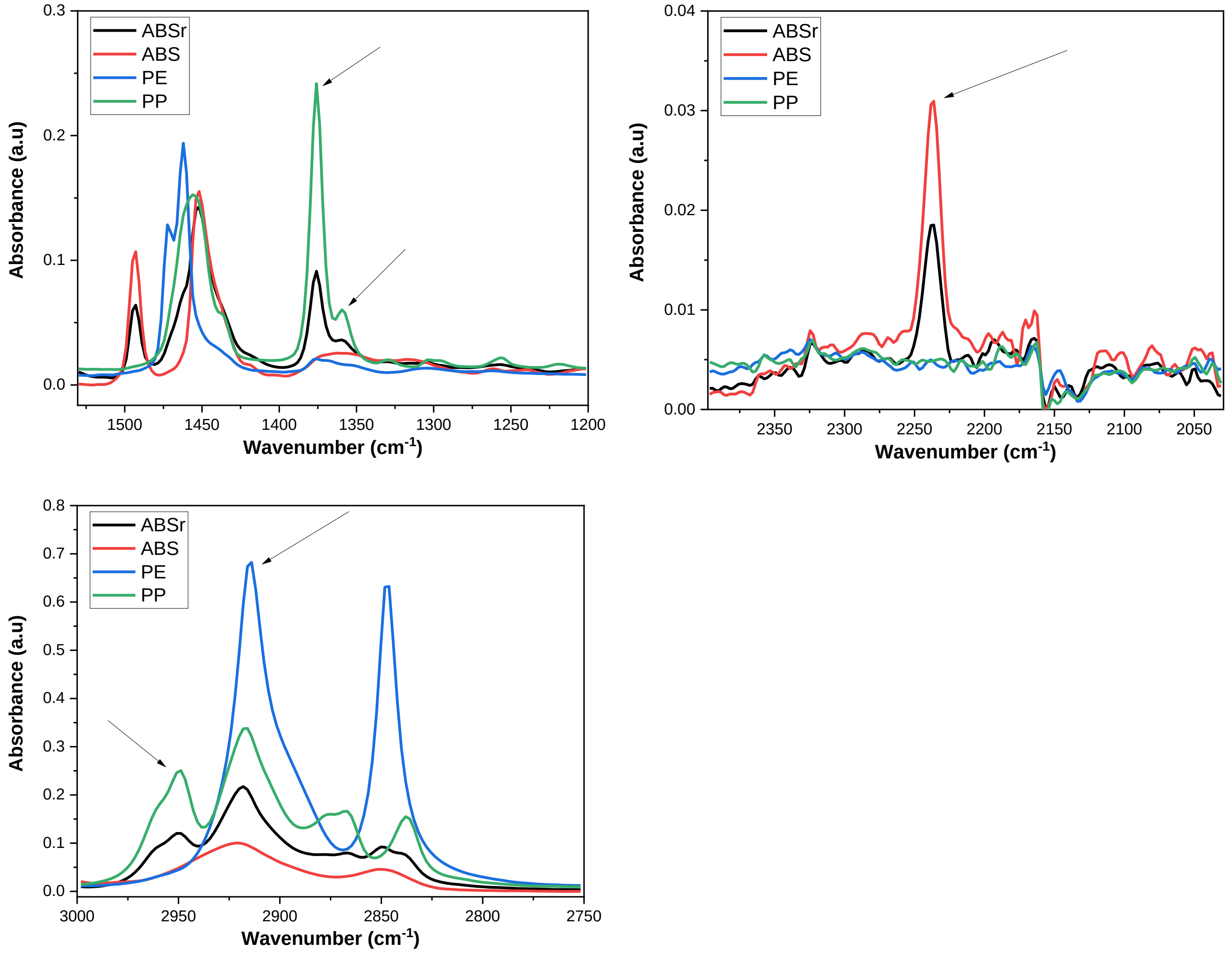

Figure S2. FTIR spectra of ABSr, commercial ABS, PP and PE.
